# Supplementary material for: Machine learning and topological data analysis identify unique features of human papillae in 3D scans
Source: Sci Rep. 2023 Dec 14;13:21529. doi: 10.1038/s41598-023-46535-9 (PMC10721919; doi:10.1038/s41598-023-46535-9)
Supplement: Supplementary file 1 — Supplementary Information. [file 41598_2023_46535_MOESM1_ESM.pdf]

Supplementary material

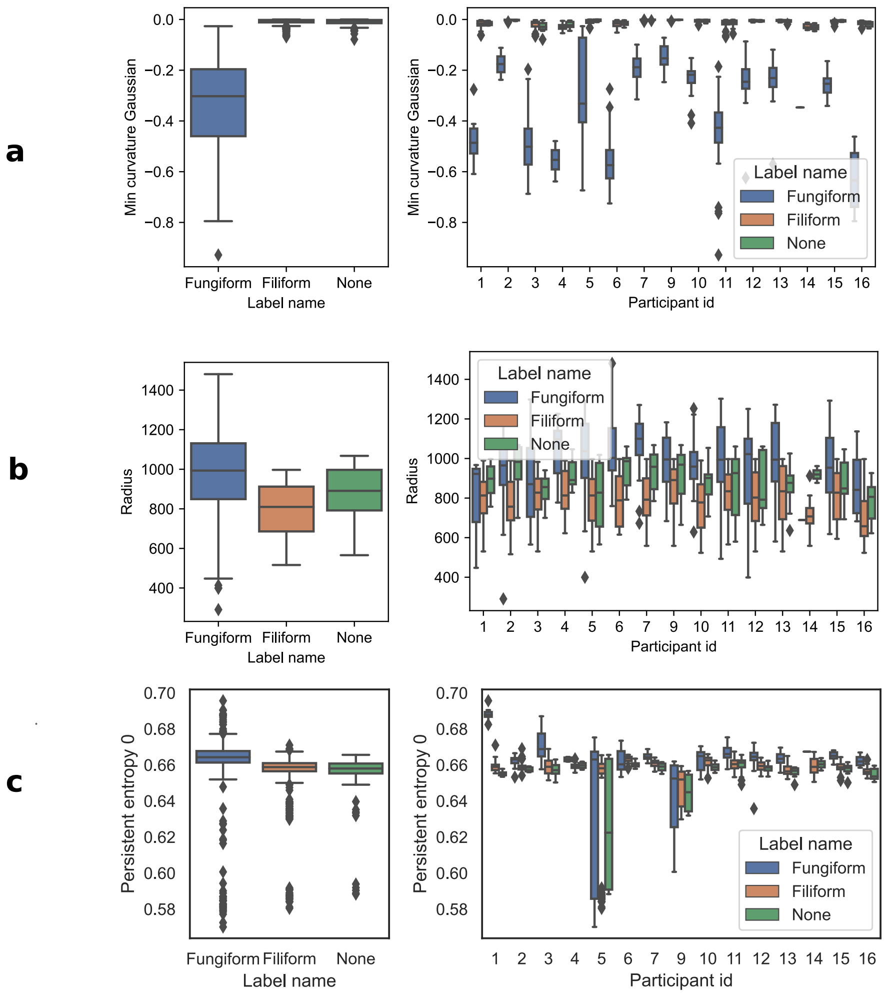

Figure S1. The most important features for papillae type classification (a-c) are the features with the highest importance for the papillae type classification task. (a) and (c) are topological, while (b) is curvature.

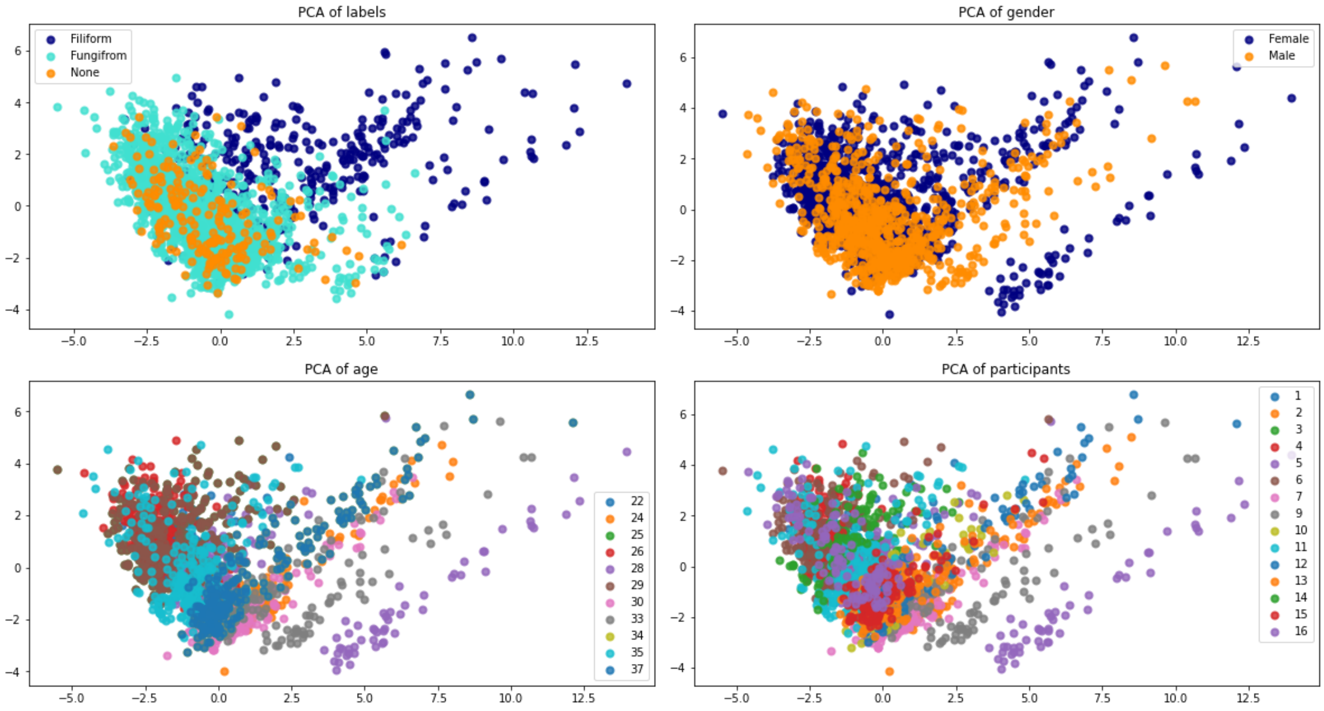

Figure S2. Here we see PCA plots for all the features. In the first row on the left, we see the labels for the 3 classes. The second plot in the first rows shows the gender. In the third plot we see the different ages of the participants. In the fourth plot we see the PCA plot for the participants. We can see that participant 9 and participant 5 are clustered very nicely. The remaining participant's features also exhibit some sort of clustering though not as clear as the others, which is an interesting observation.

Feature type	Feature name	Description	Intuitive interpretation
Baseline features	Radius	The radius of the papillae	The euclidean distance between the peak and boundary of a sphere with 90% of points.
	Height	The height of the papillae	The vertical distance between the top of the papilla and the best fit plane.
Curvature features	Minimum Gaussian	The minimum value of the Gaussian curvature	How pointy downwards an object is
	Maximum Gaussian	The maximum value of the Gaussian curvature	How pointy the object is at its maximum
	Ratio Gaussian	The ratio with (+) $k_{Gaussian}$ over (-) $k_{Gaussian}$, if # of (+) $\leq$ # (-); and the other way round	Approximately measuring the papilla curves towards the normal
	Ratio mean	The ratio with (+) k_{mean} over (-) k_{mean}, if # of (+) $\leq$ # of (-); and the other way round	Approximately measuring how positively curved the papilla is
	Positive Gaussian	The percentage of points with positive Gaussian curvature	A measure of how positively curved the papilla is, what percentage is dome-like
	Positive mean	The percentage of points with positive mean curvature	The percentage of points with positive mean curvature
Topological features	Persistent entropy (0)	The Shannon entropy of the barcode in H_0	Measuring how different the lengths of the bars are in H_0
	Short bars (0) and (1)	The number of short bars in H_0 and H_1, respectively	The number of least persistent connected components in H_0 and loops in H_1 with relatively short life span
	Amplitude (Bottleneck) (0) and (1)	The distance between the persistence diagram and the empty diagram in the Bottleneck metric	Approx. measurement of the length of the longest bar, or the life span of the most persistent feature
	Amplitude (Persistence image)	The distance between the persistence image and the empty diagram	Quantity measuring the topology of the object and how much it differs from a flat surface

Table S1. Description of non-correlated baseline, curvature and topological features.

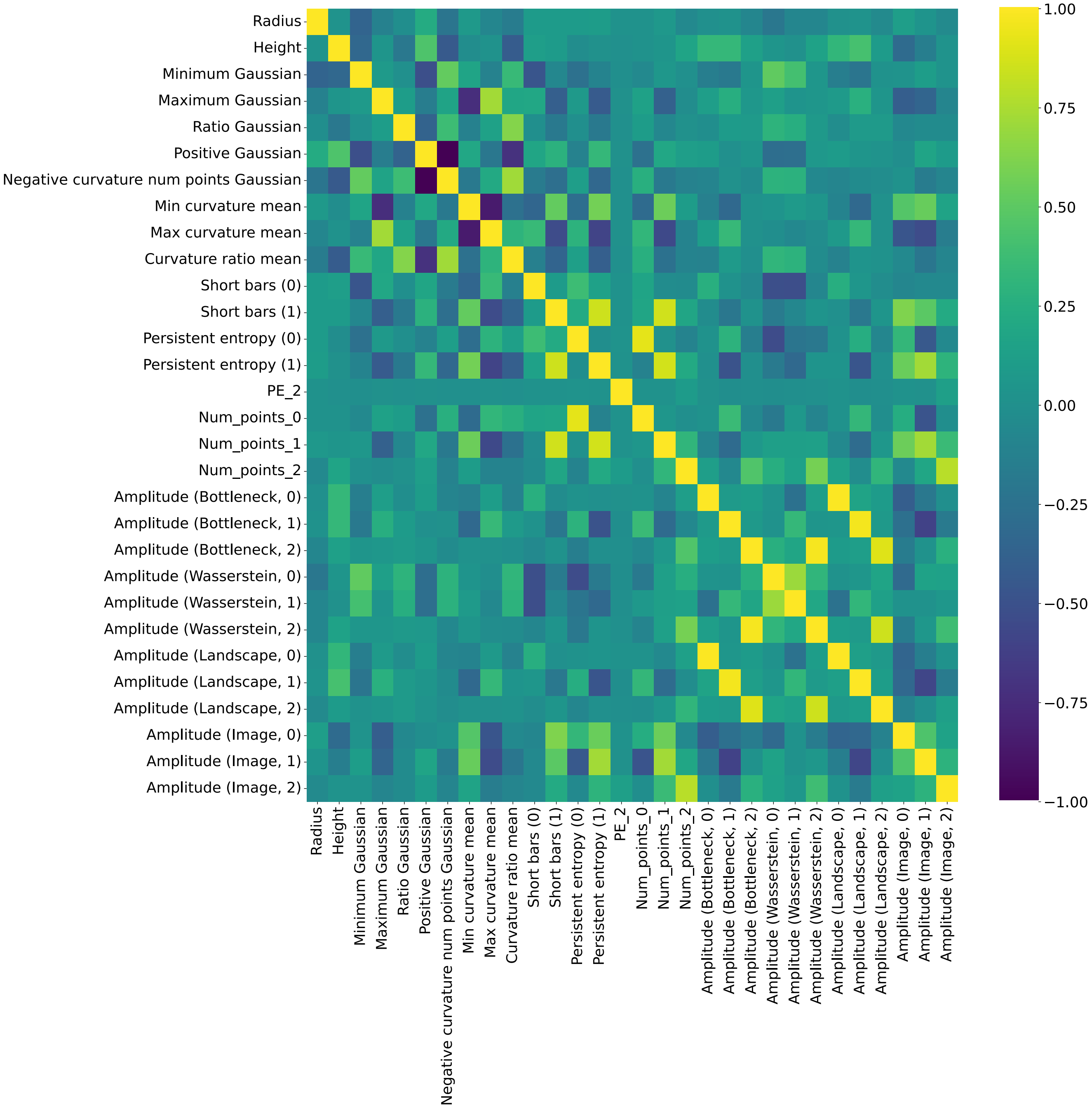

Figure S3. Correlation matrix for all features

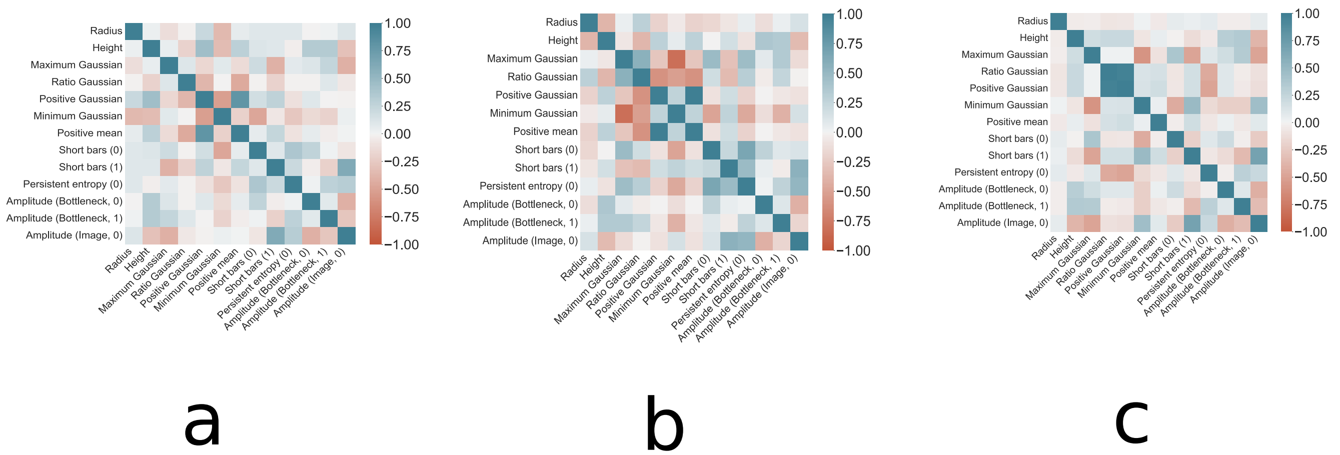

Figure S4. Correlation matrices (a) is the correlation matrix for all papillae types, (b) is the correlation matrix for fungiform and (c) is the correlation matrix for filiform. We filtered down to this set of features by removing other features that had high correlation (more than 0.65). The features remaining on this matrix show little correlation, implying that they capture different properties of papillae shape. Thus they are the basis of our analysis. However, once we filter by papillae type, some higher correlation is visible. For example, when we look at the correlations for the features of fungiform only, there is almost perfect correlation between Positive mean and Positive Gaussian. This was hidden in the overall correlation matrix.

Participant id	Segments
1	173
2	163
3	168
4	149
5	199
6	138
7	139
9	115
10	77
11	240
12	132
13	106
14	51
15	121
16	121

Table S2. Number of segments per participant.

Gender	Age (mean;SD)
Female	29.5 (4.5)
Male	28.3 (3.9)

Table S3. Demographics of the participants.

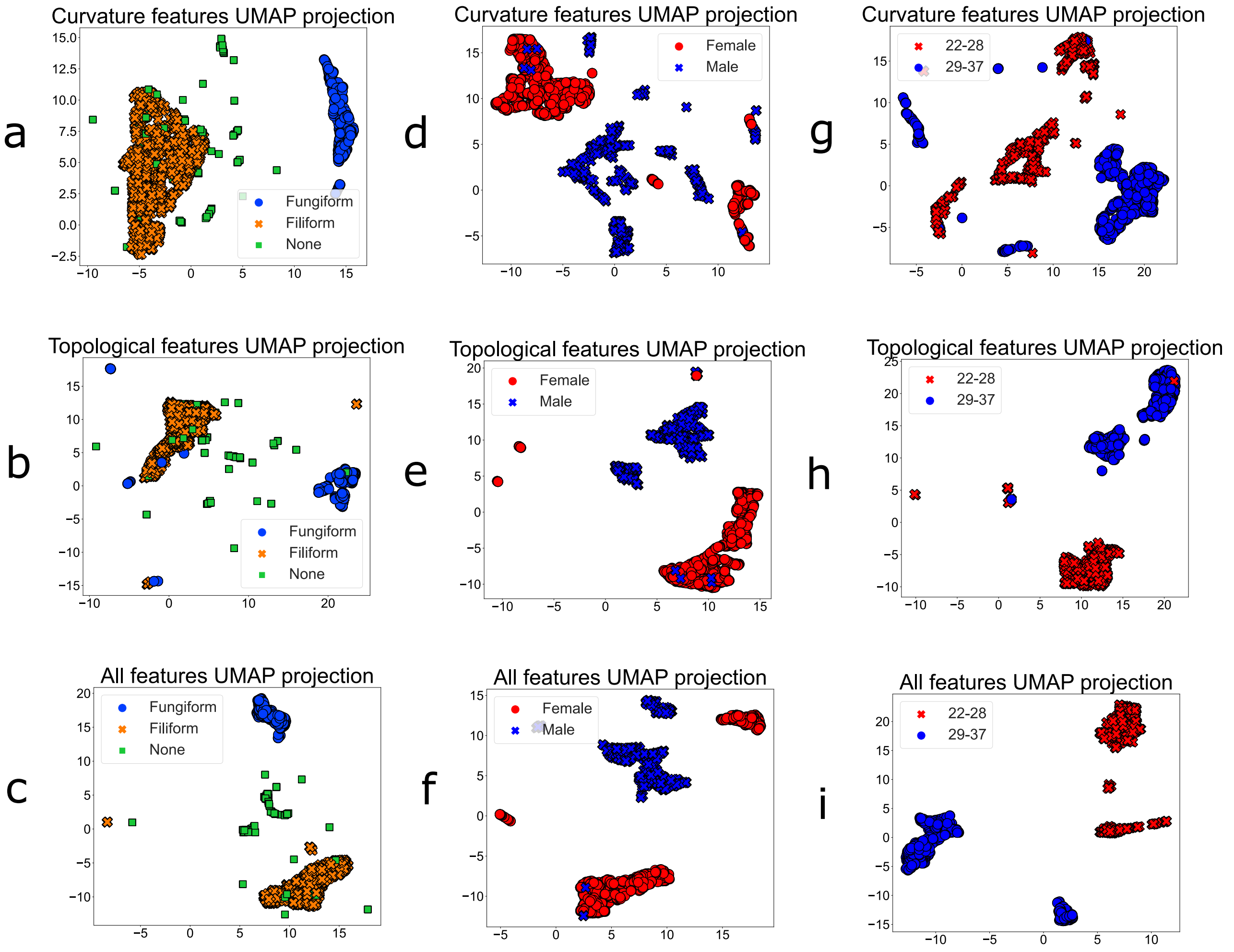

Figure S5. UMAP plots for three classification tasks. (a-c) are for papillae type, (d-f) are for gender and (g-i) are for the age task. The clusters are more well defined when we look from the top to the bottom (from curvature to all features), which is also demonstrated by the performance at the classification tasks – the topological features outperform the curvature, and the combination of all features together achieves the best performance.

Model	Balanced acc(Gender, all)	Balanced acc(Gender, lim)	Balanced acc(Age)	Balanced acc(Age,lim)
Baseline features	0.45 ± 0.12	0.52 ± 0.06	0.52 ± 0.11	0.52 ± 0.11
Curvature features	0.44 ± 0.25	0.67 ± 0.15	0.57 ± 0.30	0.59 ± 0.28
Topological features	0.45 ± 0.29	$\mathbf{0.67 \pm 0.11}$	0.57 ± 0.30	$\mathbf{0.61 \pm 0.27}$
All Combined	$\mathbf{0.42 \pm 0.28}$	0.65 ± 0.14	0.50 ± 0.24	0.53 ± 0.21

Table S4. Balanced accuracies for age and gender tasks. The performance when we use Leave-one-group-out approach. The results are worse than before due to a small number of participants having too low accuracies. It will be a question for future work to investigate this. The results on the right are when these small number of participants have been removed. It can be that they are outliers and their topological features do. However, it is difficult to make conclusions given the small sample size. It could be that some people are topological outliers, and their features are not similar at all to the other people in the same age category

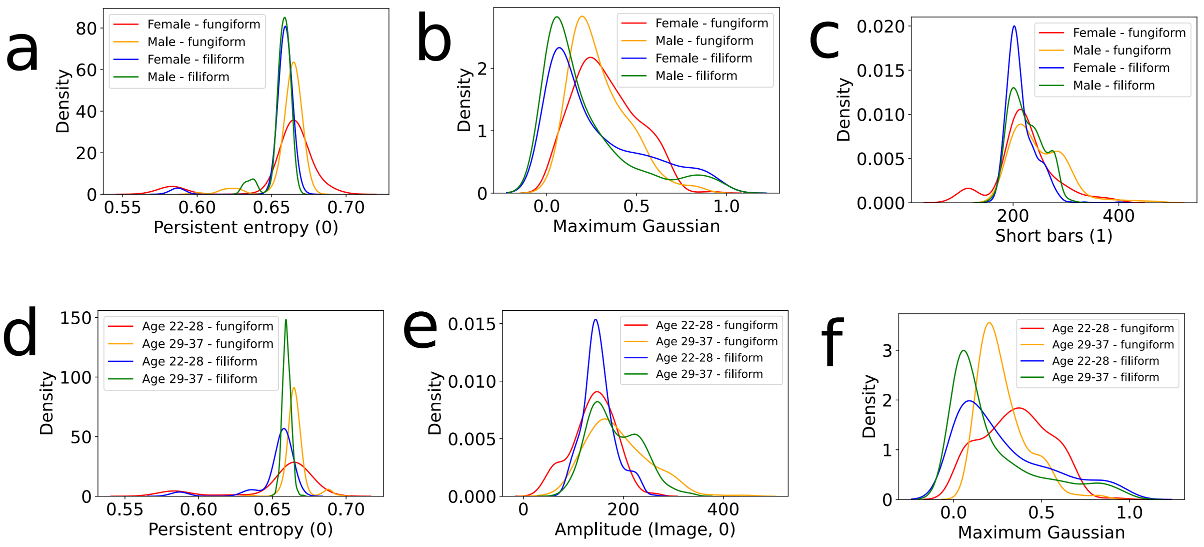

Figure S6. **Comparison between the papillae type for the most important gender and age features** .

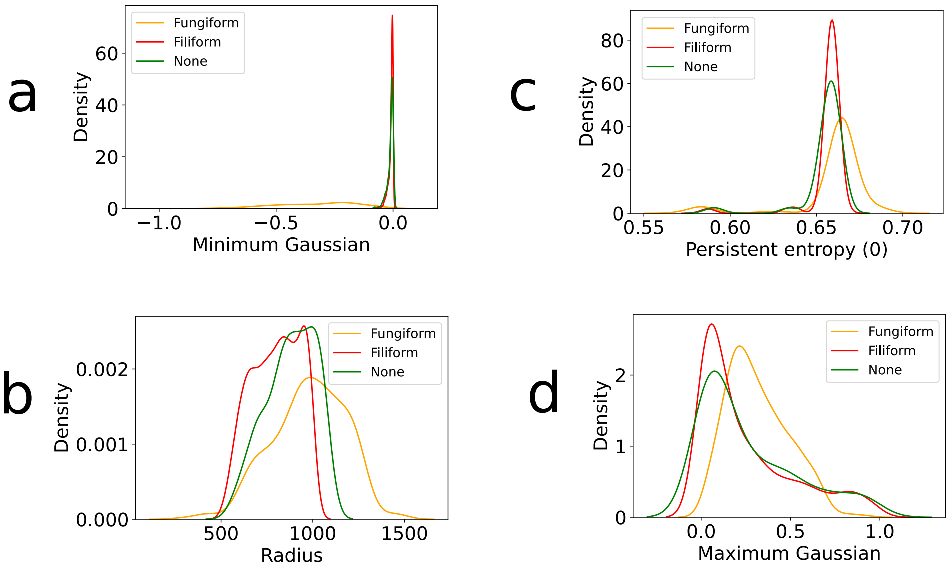

Figure S7. **KDE plots of most important features for type classification task**. In plot (a), the distribution of the Minimum Gaussian for Fungiform follows a very different pattern from Filiform and None – it is mostly flat and evenly distributed on the interval $(-1,0)$, while Filiform and None are densely concentrated around 0. In plot (b), the distribution of Filiform and None are very similar, with Fungiform having higher value of Radius, which is as expected from previous work[18]. In plot(c), the papillae Types Filiform and None follow a similar pattern to (a). Fungiform has higher value of Persistent entropy (0). In plot (d), even though Maximum Gaussian is not amongst the top three most important features, the value for Maximum Gaussian is higher for Filiform and None compared to Fungiform. This is as expected due to the sharper shape of filiform with more pronounced drop and steeper sides.

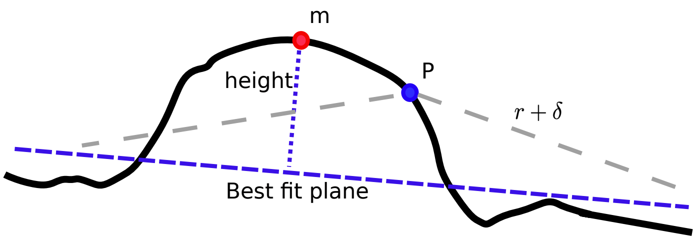

Figure S8. Schematic figure: Profile view of processing a segment with a fungiform papilla. From an arbitrary point P, all mesh vertices within a radius $r+\delta$ are taken. Then a Best fit Plane is found using the RANSAC algorithm. The candidate point for the peak of a papilla (if present) is found as m – the point furthest from the plane. This distance is taken to be the height, and m is assumed to be the centre of the papilla.
